# Metabolic Surgery Outcomes in U.S. Patients with Class I Obesity

**DOI:** 10.1089/bari.2020.0046

**Published:** 2021-06-10

**Authors:** Dustin Baldwin, Lisa Sanchez-Johnsen, Roberto Bustos, Alberto Mangano, Mario Masrur

**Affiliations:** ^1^ Department of Surgery, Division of General, Minimally Invasive and Robotics, University of Illinois at Chicago, Chicago, Illinois, USA.; ^2^ Department of Family Medicine, Rush University Medical Center, Chicago, Illinois, USA.

**Keywords:** class 1 obesity, RYGB, LSG, metabolic surgery, BMI less than 35

## Abstract

***Background:*** Although numerous studies outside the United States (U.S.) have explored weight loss and comorbidity resolution among patients with class I obesity (body mass index [BMI] 30–34.9 kg/m^2^) after metabolic surgery, few U.S.-based studies have been conducted.

***Objective:*** Our aim was to compare weight loss and comorbidity resolution among U.S. patients with class I obesity, who underwent laparoscopic sleeve gastrectomy (LSG) versus Roux-en-Y gastric bypass (RYGB). ***Methods:*** Weight loss and comorbidity data among only patients with class I obesity, who underwent LSG or RYGB, were examined. Between April 2009 and April 2017, 1215 metabolic surgeries were performed with 30 patients meeting the inclusion criteria (17 LSG and 13 RYGB).

***Results:*** Percent total weight loss (%TWL) for LSG peaked at 12 months (20.85%), while RYGB %TWL peaked at 18 months (21.65%). Percent excess weight loss (%EWL) peaked at 12 months after LSG (83.59%) and 18 months after RYGB (98.29%). Overall follow-up was 56.3%, 36.7%, and 43.3% at 12, 18, and 24 months. LSG and RYGB were both successful with regard to resolution of medical comorbidities at 12 months.

***Conclusion:*** RYGB and LSG appear to have similar, successful outcomes among U.S. patients with class I obesity for weight loss and comorbidity resolution.

Obesity rates continue to rise from 30.5% in the year 2000 to 39.8% among U.S. adults in the years 2015–2016.^1^ In addition, between the years of 1999–2002 and 2011–2014, the percentage of adults with class I obesity (body mass index (BMI) 30–34.9 kg/m^2^) increased from 17.9% to 20.6%, with more than half of those who are obese falling into the class 1 obesity range.^2^ All classes of obesity, including class I, are associated with increased risk of hypertension, diabetes, obstructive sleep apnea (OSA), and other obesity-related comorbidities.^2^ Numerous benefits have been attributed to metabolic surgery, including resolution of diabetes,^3,4^ hypertension,^3^ hyperlipidemia,^4^ gastroesophageal reflux (GERD),^5^ and OSA.^6^

Despite the established benefits of metabolic surgery, particularly laparoscopic sleeve gastrectomy (LSG) and Roux-en-Y gastric bypass (RYGB), these surgeries are traditionally performed in patients with BMI >40.0 kg/m^2^ regardless of comorbidities and in patients with BMI >35.0 kg/m^2^ with at least one comorbidity related to obesity.^7^ These guidelines were established at the 1991 National Institutes of Health (NIH) consensus conference.^8^ As these recommendations are nearly 30 years old, they warrant a review based on the mounting experience and outcomes from bariatric surgery and improved surgical technology. Recently, the American Society for Metabolic and Bariatric Surgery (ASMBS) recommended metabolic surgery should be offered as an option for suitable individuals with BMI 30–34.9 kg/m^2^ and obesity-related comorbidities (especially type 2 diabetes), who have not achieved substantial, durable weight loss and comorbidity improvement with reasonable nonsurgical methods.^9^ The 2016 joint statement by International Diabetes Organizations stated metabolic surgery should be recommended to treat type 2 diabetes in patients with class II and III obesity. Surgery should also be considered for patients with type 2 diabetes and class I obesity if hyperglycemia is inadequately controlled despite optimal treatment with either oral or injectable medications.^10^

Due to the stringent eligibility criteria outlined above, metabolic surgery has been performed on patients with class I obesity primarily outside of the United States. Several large randomized and observational studies examining metabolic surgery in class I obesity have been performed in Chile,^5,11^ Brazil,^12^ Lebanon,^13^ and India.^14^ These studies revealed positive results with regard to weight loss and comorbidity resolution. Despite the success of metabolic surgery among those with a BMI less than 35 kg/m^2^, there have been only a few studies (mainly laparoscopic adjustable gastric banding [LAGB] and gastric balloons) that have been conducted within the United States with these patients.^15–18^ As the United States is a racial and ethnically diverse population, international studies may not be generalizable to a U.S population of patients with class I obesity. Thus, there is a continued need to examine the outcomes of metabolic surgery, specifically RYGB and LSG, in U.S. patients with BMI less than 35 kg/m^2^.

At our institution, patients with BMIs less than 35 kg/m^2^ have undergone LSG or RYGB, for various indications. The indications for surgery included patients with a BMI greater than 35 kg/m^2^ during the preoperative multidisciplinary evaluation, but on the day of surgery lost weight to achieve a BMI less than 35 kg/m^2^. Several patients had other indications for metabolic surgery, including a patient with LAGB slippage requiring revisional surgery, two patients with severe GERD requiring conversion of LSG to RYGB, and one patient with revisional surgery for insufficient weight loss. Therefore, the primary aim of this study was to compare weight loss and BMI changes at specific time points among U.S. patients with a BMI less than 35 kg/m^2^, who underwent LSG versus RYGB. The secondary aim was to examine the resolution versus nonresolution of medical comorbidities—specifically diabetes, hypertension, hyperlipidemia, GERD, and OSA post-RYGB or post-LSG among these patients.

## Methods

This is a retrospective review of a prospectively maintained database. Inclusion criteria were as follows: all patients who underwent metabolic surgery with a BMI less than 35 kg/m^2^ on the day of surgery, during the time period April 2009–April 2017. Patients must have undergone exclusively LSG or RYGB. Finally, patients must have reached at least 2 years postsurgery follow-up at the time of data collection. Exclusion criteria were any patient with BMI greater than 35 kg/m^2^ on day of surgery, any patient who underwent LAGB or gastric balloon, or any patient with less than 2-year follow-up.

Data were extracted from electronic medical records and included the following demographic and surgical variables: age, race, sex, weight, BMI and type of bariatric surgery. Presurgical and postsurgical comorbidities (diabetes, hypertension, hyperlipidemia, GERD, and OSA) were also extracted. Two team members independently reviewed and double checked all medical records for accuracy. As this study was retrospective, data were collected on every patient who met inclusion criteria. Therefore, the sample size was limited to 30 patients. This study was conducted with approval from the University of Illinois at Chicago Institutional Review Board (IRB protocol #2011–1104).

Postoperative comorbidities were defined according to the ASMBS outcome reporting standards.^9^ “Remission” and “improvement” indicated the comorbidity had completely cured (off all medications) or improved (decrease in medication) from baseline. “Unchanged” or “worsened” meant the patient was on the same medication profile (unchanged) or had increased medications (worsened). For purposes of this article, remission and improvement were combined into a category called “Resolved.” If the patient remained unchanged or worsened, they were considered “Nonresolved.” Individual clinic visits, medications, and laboratory values were reviewed to determine ASMBS outcome reporting standards. Due to low sample numbers, comorbidities were not compared directly. Instead, we performed a descriptive analysis of comorbidity resolution after LSG and RYGB rather than a comparative analysis.

For weight loss and BMI analyses, the ASMBS Standardized Outcomes Reporting in Metabolic and Bariatric Surgery was followed reporting change in BMI, percent total weight loss (%TWL), and percent excess weight loss (%EWL).^9^ Percent TWL, %EWL, and change in BMI for RYGB and LSG patients were calculated at 1-, 6-, 12-, 18-, and 24-month follow-up and compared using a Student's *t*-test with statistical significance set at *p* < 0.05. We had sufficient data on %TWL, %EWL, and change in BMI to perform statistical analysis, compared to our comorbidity analysis, which we left as descriptive due to low numbers of individual comorbidities among patients.

## Results

Between April 2009 and April 2017, 1215 primary metabolic surgeries (334 RYGB and 881 LSG) were performed at our institution. Of those, 30 patients met the inclusion criteria with 17 undergoing LSG and 13 undergoing RYGB. All preoperative demographic, metabolic, and comorbidity values, along with associated *p*-values are listed in Table 1. Overall postsurgical follow-up are as follows: 100% at 1 month, 80.0% at 6 months, 56.7% at 12 months, 36.7% at 18 months, and 43.3% at 24 months. Please see Table 2 for full details.

**Table 1.** Preoperative Variables

N	LSG			RYGB			Overall		
	17			13			30		
	Mean	Range	SD	Mean	Range	SD	Mean	p-value	SD
Demographics									
Age (years)	47.7	(30–65)	(10)	50.8	(30–67)	(9.3)	49.1	0.39	(9.8)
BMI (kg/m^2^)	33.8	(30.2–34.9)	(1.2)	32.7	(30.3–34.7)	(2.1)	33.3	0.08	(1.7)
Weight (kg)	91.1	(75.4–101.0)	(5.4)	89.9	(68.4–105.8)	(9.8)	90.6	0.67	(8.9)

	(*n*)	(% of LSG)		(*n*)	(% of RYGB)		(*n*)	p-value	(Overall %)
Comorbidities (*n*)									
T2D	6	35.30%		6	46.20%		12	0.71	40.00%
HTN	7	41.20%		7	53.80%		14	0.71	46.70%
HLD	7	41.20%		6	46.20%		13	1.0	43.30%
GERD	4	23.50%		6	46.20%		10	0.26	33.30%
OSA	4	23.50%		1	7.70%		5	0.36	16.70%
Race/Ethnicity (*n*)									
Hispanic	6	35.30%		5	38.50%		11	1.0	36.70%
White	3	17.60%		3	23.10%		6	1.0	20.00%
Black	8	47.10%		5	38.50%		13	0.72	43.30%

Weight listed is on day of surgery for LSG, RYGB, and overall. Preoperative variables compared using a Student's *t*-test with statistical significance set at *p* < 0.05.

N, sample size; BMI, body mass index; SD, standard deviation; kg, kilogram; RYGB, Roux-en-Y gastric bypass; LSG, laparoscopic sleeve gastrectomy, T2D, type 2 diabetes mellitus; HTN, hypertension; HLD, hyperlipidemia; OSA, obstructive sleep apnea; GERD, gastroesophageal reflux disease.

**Table 2.** Postsurgery Results for Body Mass Index and Weight Loss

	Postsurgery follow-up time periods				
	1 month	6 months	12 months	18 months	24 months
Change in BMI					
Mean (SD)					
LSG	2.00 (1.01)	6.25 (2.35)	6.98 (4.89)	5.82 (5.32)	5.54 (3.31)
RYGB	1.71 (0.89)	5.83 (1.89)	7.11 (2.34)	7.10 (3.08)	6.08 (4.46)
*p*-value	0.44	0.66	0.94	0.66	0.82
BMI (kg/m^2^)					
Mean (SD)					
LSG	31.79 (1.34)	27.41 (3.03)	26.74 (4.89)	27.87 (5.87)	28.30 (3.80)
RYGB	30.98 (1.88)	26.56 (1.55)	25.39 (1.65)	24.81 (1.60)	25.41 (3.61)
*p*-value	0.19	0.43	0.45	0.30	0.24
% Total weight loss					
Mean (SD)					
LSG	5.90 (2.91)	18.69 (7.38)	20.85 (13.52)	17.53 (16.13)	16.51 (10.12)
RYGB	5.19 (2.59)	17.79 (5.21)	21.58 (6.55)	21.65 (8.93)	18.65 (13.46)
*p*-value	0.51	0.75	0.89	0.64	0.77
% Excess weight loss					
Mean (SD)					
LSG	22.94 (11.77)	75.76 (37.81)	83.59 (58.58)	72.07 (69.37)	65.41 (45.34)
RYGB	23.52 (13.27)	80.72 (24.55)	96.37 (20.90)	98.29 (23.24)	84.38 (49.80)
*p*-value	0.90	0.72	0.55	0.45	0.53
Patient follow-up					
*N* (%)					
LSG	17 (100.0%)	13 (76.5%)	6 (35.3%)	5 (29.4%)	8 (47.1%)
RYGB	13 (100.0%)	11 (84.6%)	11 (84.6%)	6 (46.2%)	5 (38.5%)
Overall	30 (100.0%)	24 (80.0%)	17 (56.7%)	11 (36.7%)	13 (43.3%)

Table lists change in BMI, BMI, % total weight loss, % excess weight loss, and patient follow-up at 1-, 6-, 12-, 18-, and 24-month follow-up time periods. The *p*-value at each time point compares LSG to RYGB at separate time points (1, 6, 12, 18, and 24 months) with respect to change in BMI, BMI, %TWL, and %EWL. Significance is defined as *p*-value <0.05. Follow-up percentages are compared to the sample size at baseline.

SD, standard deviation; N, number.

**FIG. 1.**
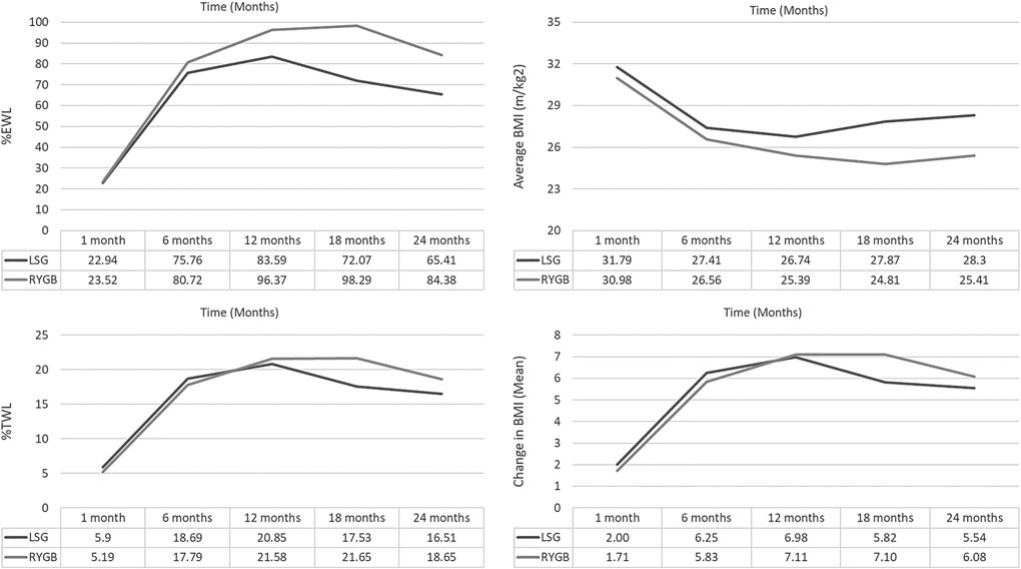
Representative graphs comparing LSG to RYGB with regard to percent excess weight loss (%EWL), percent total weight loss (%TWL), average BMI, and change in BMI at 1-, 6-, 12-, 18-, and 24-month follow-up time periods. RYGB, Roux-en-Y gastric bypass; LSG, laparoscopic sleeve gastrectomy.

As seen in Table 2 and Figure 1, when comparing LSG to RYGB at various follow-up time points, there was no significant difference between either operation with regard to change in BMI, %TW or %EWL (all *p*-values >0.05). At the 12-month follow-up, patients who underwent RYGB had a %EWL of 96.37% compared to a %EWL of 83.59% for LSG. The %EWL peaked at 12 months after LSG (83.59%) and at 18 months after RYGB (98.29%).

A visual inspection of the means revealed that patients who underwent LSG achieved their largest %EWL, %TWL, and BMI improvements at 12 months, while patients who underwent RYGB had their largest improvements at 18 months. When comparing both procedures, although there was a larger change in BMI, %TWL, and %EWL in the RYGB versus the LSG group at every follow-up time point, there was no significant difference between the groups (all *p*-values >0.05).

In general, a %TWL of >20% can be used to define successful weight loss.^19^ At 12 months, patients undergoing both LSG (20.85%) and RYGB (21.58%) achieved successful weight loss using this definition. Results also revealed that patients who underwent LSG peaked at 12 months and by the 18- and 24-month follow-up, their %TWL decreased to 17.53% and 16.51%, respectively. The %TWL among patients who underwent RYGB peaked at 18 months (21.65%) with decrease to 18.65% at 24 months.

Comorbidity resolution was examined at 12 months due to the fewer number of patients who attended the subsequent follow-up time periods. We performed a descriptive analysis of comorbidity resolution as our numbers precluded comparative analysis. Postoperative resolution of individual comorbidities at 12 months is shown in Table 3. At 12 months postsurgery, diabetes was resolved in all patients who had diabetes presurgically. Hypertension, hyperlipidemia, and OSA had higher percentage of resolution after RYGB based on a visual inspection of the means.

**Table 3.** Postsurgery Resolution of Medical Comorbidities at 12 Months

Comorbidities at 12 months postsurgery		
	LSG	RYGB
Diabetes (*N*)		
*N* (%)		
Preoperative	6	6
Resolved	6 (100.0%)	6 (100.0%)
Nonresolved	0 (0.0%)	0 (0.0%)
Hypertension (*N*)		
*N* (%)		
Preoperative	7	7
Resolved	2 (28.6%)	6 (85.7%)
Nonresolved	5 (71.4%)	1 (14.3%)
Hyperlipidemia (*N*)		
*N* (%)		
Preoperative	7	6
Resolved	3 (42.9%)	5 (83.3%)
Nonresolved	4 (57.1%)	1 (16.7%)
GERD (*N*)		
*N* (%)		
Preoperative	4	6
Resolved	0 (0.0%)	4 (66.7%)
Nonresolved	5^*^ (100.0%)	2 (33.3%)
OSA (*N*)		
*N* (%)		
Preoperative	4	1
Resolved	1 (25.0%)	1 (100.0%)
Nonresolved	3 (75.0%)	0 (0.0%)

Preoperative, resolved, and nonresolved comorbidities are listed above. Numbers listed are number of patients within each surgical category, with percentages listed simultaneously. Preoperative is defined as day of surgery. Resolved and nonresolved comorbidities are listed at 12 months.

LSG, laparoscopic sleeve gastrectomy; RYGB, Roux-en-Y gastric bypass; GERD, gastroesophageal reflux disease; OSA, obstructive sleep apnea; N, number of patients.

^*^ One patient developed GERD postoperatively.

Patients who had preoperative GERD had improvement post-RYGB; whereas none of the patients who underwent LSG had resolution of GERD. Moreover, one patient without preoperative GERD developed GERD after LSG.

## Discussion

Metabolic surgery has been shown to improve weight loss and medical comorbidities compared to pharmacological treatment of obesity, even among patients with class I obesity.^20^ However, there is a paucity of data from the United States, as most surgery for patients with class I obesity comes from South America, Asia, or Europe. We complied an abbreviated sample of studies, both in the United States and worldwide, including patients with class I obesity who underwent metabolic surgery (Table 4). Our study, although with a small cohort, adds to the growing body of literature regarding the benefits of metabolic surgery among patients with class I obesity. Moreover, this study reports no significant difference between RYGB and LSG in terms of weight loss or BMI resolution. It is also one of the largest studies from the United States on patients with class I obesity undergoing metabolic surgery, particularly LSG and RYGB. The lack of U.S data can be partially explained by insurance companies not covering bariatric surgery for patients with a BMI below 35 kg/m^2^, except for LAGB, and in some states, gastric balloons. The challenge associated with conducting metabolic surgery among patients with BMI less than 35 kg/m^2^ in the United States makes this article unique. Finally, to our knowledge, this is the first direct comparison between LSG and RYGB in terms of weight loss and BMI resolution in strictly U.S. patients with class I obesity.

**Table 4.** Summary of Selected Studies in Both the United States and Worldwide Examining Patients with Body Mass Index Less Than 35, Who Underwent Bariatric Surgery

Author, Year	Country	(*n*)	BMI range	Procedure
Angrisani *et al*.,^24^ 2004	Italy	225	<35	LAGB
Parikh *et al*.,^17^ 2006	USA	93	30–35	LAGB
Dixon *et al*.,^21^ 2008	Australia	60	30–40	LAGB vs. Medical
Sultan *et al*.,^16^ 2009	USA	53	<35	LAGB
DeMaria *et al*.,^15^ 2010	USA	218	<35	LAGB vs. RYGB
Scopinaro *et al*.,^25^ 2011	Italy	30	<35	BPD
Cohen *et al*.,^12^ 2012	Brazil	66	<35	RYGB
Lee *et al*.,^26^ 2014	Taiwan	60	25–35	LSG vs. LAGB
Parikh *et al*.,^18^ 2014	USA	57	30–35	BS vs. Medical
Ikramuddin *et al*.,^27^ 2015	USA/Taiwan	36	30–40	RYGB vs. Medical
Hsu *et al*.,^28^ 2015	Taiwan	52	<35	LSG/RYGB vs. Medical
Maiz *et al*.,^11^ 2015	Chile	1119	<35	LSG vs. RYGB
Noun *et al*.,^13^ 2016	Lebanon	541	30–35	LSG
Kular *et al*.,^14^ 2016	India	128	30–35	MGB
Murad *et al*.,^29^ 2017	Brazil	102	30–35	RYGB
Berry *et al*.,^3^ 2018	Chile	252	30–35	LSG
Amin *et al*.,^30^ 2019	Pakistan	209	30–35	LSG vs. Medical

Table not meant to be all inclusive of every study, rather a representation of selected studies. Studies are listed with author, year, country of origin, number of patients in the study, and BMI range of patients.

LAGB, laparoscopic adjustable gastric banding; BS, bariatric surgery; BPD, biliopancreatic diversion, RYGB, Roux-en-Y gastric bypass; LSG, laparoscopic sleeve gastrectomy; MGB, mini/one anastomosis gastric bypass.

As seen in Table 4, most studies examining metabolic surgery among patients with BMI less than 35 kg/m^2^ were conducted outside of the United States. Maiz *et al.*^11^ examined 1119 Chilean patients with BMI less than 35 kg/m^2^, who underwent metabolic surgery (LSG or RYGB). Data at 12 months showed improvements in BMI from 33.1 kg/m^2^ to 24.5 kg/m^2^ and %EWL of 107.9%. Noun *et al.*^13^ examined 541 Lebanese patients who underwent LSG with a BMI of 30–35 kg/m^2^. Average BMI improved from a preoperative value of 32.59 kg/m^2^ to 24.7, 24.9, and 26.4 kg/m^2^ at 1, 2, and 5 years, respectively. In another study, Berry and colleagues^3^ examined 252 Chilean patients with a BMI less than 35 kg/m^2^, who underwent LSG. BMI improved from 32.3 kg/m^2^ at presurgery to 22.97 kg/m^2^ at 12 months postsurgery with %EWL of 98.42% at 12 months postsurgery. In our study at 12 months follow-up, patients who underwent RYGB had a %EWL of 96.37% compared to a %EWL of 83.59% for LSG. The %EWL peaked at 12 months after LSG (83.59%) and at 18 months after RYGB (98.29%).

In one of the few U.S.-based studies examining metabolic surgery in class I obesity, Parikh *et al.*^18^ reported findings of 57 U.S. patients with BMI 30–35 kg/m^2^ randomized to medical management versus bariatric surgery. Of the 28 patients who were in the surgical arm, 17% underwent LAGB, 55% underwent LSG, and 24% underwent RYGB. At 6 months, surgical patients reported a significantly greater %EWL and change in BMI versus medical patients. In another study, DeMaria *et al.*^15^ utilized a national database to identify 235 U.S. patients with BMI 30–35 kg/m^2^, who underwent bariatric surgery (LAGB vs. RYGB). Of the 235 patients, 50% underwent LAGB and 50% underwent RYGB. At 12 months, RYGB patients achieved a greater decrease in their postoperative BMI compared to LAGB (27.1–30.9 kg/m^2^). Finally, one study examined 53 class I obesity U.S. patients who underwent LAGB and found a mean BMI decrease from 33.1 preoperatively to 28.1, 25.8, and 25.8 kg/m^2^ at 6, 12, and 24 months postsurgery.^16^

Outside of the United States, large randomized trials^21^ have examined LAGB in patients with BMIs less than 35 kg/m^2^. These studies reported favorable outcomes in class I obese patients undergoing LAGB compared to medical management.^21^ However, LAGB has fallen out of favor in the past decade, compared to LSG and RYGB, with band slippage and weight loss failure reported at nearly 50%.^22^ Furthermore, resolution of comorbidities is lower with LAGB, along with worsening of GERD.^22^ In our cohort, LAGB patients were excluded.

Several international studies have examined RYGB in diabetic patients with class I obesity. Cohen *et al.*^12^ followed 66 diabetic, class I obese patients who underwent RYGB for 6 years. Results revealed diabetes remission occurred in 88% of cases, along with remission or improvement in hypertension, hyperlipidemia, hypertriglyceridemia, and 10-year cardiovascular risk assessment. In Taiwan, a randomized controlled trial comparing LSG versus RYGB among patients with poorly controlled diabetes and BMI 25–35 kg/m^2^ revealed diabetes was resolved in 93% of RYGB patients and 47% of LSG patients at 1 year.^23^

A systemic review of bariatric surgery in patients with BMI less than 35 kg/m^2^ concluded that diabetic patients with class I obesity achieved greater improvements in weight loss, diabetes, blood pressure, and hyperlipidemia after bariatric surgery than nonsurgical interventions.^16^ Furthermore, results from the 5-year, prospective randomized STAMPEDE trial demonstrated that metabolic surgery (RYGB or LSG) plus intensive medical therapy (IMT) was more effective than IMT alone in decreasing hyperglycemia in U.S. patients with a BMI of 27 kg/m^2^ and greater.^4^ The results of these studies coincide with our study, which demonstrated a 12-month resolution of diabetes in all patients regardless of RYGB or LSG.

Few articles have examined hypertension and hyperlipidemia in a strictly U.S. cohort of patients with BMI less than 35 kg/m^2^. In our small cohort of class I obese U.S. patients, both RYGB and LSG were able to resolve hypertension and hyperlipidemia, although at different percentages. Metabolic surgery should be considered for patients with class I obesity and uncontrolled hypertension or hyperlipidemia.

Numerous population-based studies support the association between obesity and GERD.^5^ GERD has typically been treated with lifestyle change and medications, with surgery being reserved for refractory cases. A systematic review identified eight studies that evaluated GERD symptoms after RYGB.^5^ All studies, but one showed an improvement in GERD symptoms after RYGB. In our cohort, GERD was resolved in all patients who underwent RYGB and none of the patients who underwent LSG. In fact, one additional patient developed GERD after LSG. In patients with significant GERD, it may be clinically prudent to consider RYGB before LSG among patients with a BMI less than 35 kg/m^2^.

### Limitations

There are several limitations to this study. First, this was a retrospective and nonrandomized trial. Second, this study included a small cohort of 30 U.S. patients further divided into LSG or RYGB. Also, there were several patients lost to follow-up at 12, 18, and 24 months. The small sample size and follow-up rates restricted the strength of our statistical analyses. Furthermore, the statistical analysis of comorbidities was limited by the sample size, precluding a comparative analysis. These limitations notwithstanding, this study demonstrated successful outcomes and no significant difference between RYGB and LSG with regard to weight loss and BMI resolution among U.S. patients with class I obesity.

## Conclusion

Metabolic surgery has established its effectiveness among patients with class I obesity through multiple international studies. Similarly, we show metabolic surgery to be successful for class I obesity among U.S. patients. To our knowledge, this is the first direct comparison between LSG and RYGB in terms of weight loss and BMI resolution in strictly U.S. patients with class I obesity. Results revealed that there were no significant differences in %EWL, %TWL, or change in BMI between RYGB and LSG. Similarly, RYGB and LSG both yielded resolution of diabetes, hypertension, hyperlipidemia, OSA, and GERD to varying degrees. Our study adds to the mounting evidence for expansion of metabolic surgery indications to include class I obese patients.
